# Observed intra-cluster correlation coefficients in a cluster survey sample of patient encounters in general practice in Australia

**DOI:** 10.1186/1471-2288-4-30

**Published:** 2004-12-22

**Authors:** Stephanie A Knox, Patty Chondros

**Affiliations:** 1 AIHW General Practice Statistics and Classification Unit, The University of Sydney, Sydney, Australia; 2 Department of General Practice, The University of Melbourne, Melbourne, Australia

## Abstract

**Background:**

Cluster sample study designs are cost effective, however cluster samples violate the simple random sample assumption of independence of observations. Failure to account for the intra-cluster correlation of observations when sampling through clusters may lead to an under-powered study. Researchers therefore need estimates of intra-cluster correlation for a range of outcomes to calculate sample size. We report intra-cluster correlation coefficients observed within a large-scale cross-sectional study of general practice in Australia, where the general practitioner (GP) was the primary sampling unit and the patient encounter was the unit of inference.

**Methods:**

Each year the Bettering the Evaluation and Care of Health (BEACH) study recruits a random sample of approximately 1,000 GPs across Australia. Each GP completes details of 100 consecutive patient encounters. Intra-cluster correlation coefficients were estimated for patient demographics, morbidity managed and treatments received. Intra-cluster correlation coefficients were estimated for descriptive outcomes and for associations between outcomes and predictors and were compared across two independent samples of GPs drawn three years apart.

**Results:**

Between April 1999 and March 2000, a random sample of 1,047 Australian general practitioners recorded details of 104,700 patient encounters. Intra-cluster correlation coefficients for patient demographics ranged from 0.055 for patient sex to 0.451 for language spoken at home. Intra-cluster correlations for morbidity variables ranged from 0.005 for the management of eye problems to 0.059 for management of psychological problems. Intra-cluster correlation for the association between two variables was smaller than the descriptive intra-cluster correlation of each variable. When compared with the April 2002 to March 2003 sample (1,008 GPs) the estimated intra-cluster correlation coefficients were found to be consistent across samples.

**Conclusions:**

The demonstrated precision and reliability of the estimated intra-cluster correlations indicate that these coefficients will be useful for calculating sample sizes in future general practice surveys that use the GP as the primary sampling unit.

## Background

Cluster sample study designs are a cost-effective way of sampling difficult to reach populations. Examples include sampling schools to obtain cluster samples of students or medical practitioners to sample patients[1]. Cluster samples violate the simple random sample assumption of independence of observations, since observations are sampled from within the selected cluster – defined as the primary sampling unit. Observations within a cluster may be more alike than observations across clusters. This intra-cluster correlation leads to increased variation between clusters compared to the variation within clusters. Failure to account for intra-cluster correlation when designing a study where participants are recruited within clusters will lead to an under-powered study. To allow for any loss in power and precision, a cluster sample requires a larger sample size to answer the same research question as a study using simple random sampling [2-4].

Both the size of the intra-cluster correlation and the number of observations sampled within each cluster influence the power of the study. Even for a small intra-cluster correlation, as is often found in general practice and community samples, the loss of power can be appreciable, particularly if the size of the cluster is large[1,4].

Estimates of the size of intra-cluster correlations come from post hoc examination of studies that have used either allocation or sampling by cluster and a number of intervention studies have published observed intra-cluster correlation coefficients [4-6]. Many intervention studies however, still fail to report intra-cluster correlation coefficients[7] and there is even less information reported on survey studies that employ a cluster sample[1,8]. The lack of published estimated intra-cluster correlations continues to hamper the design of studies that employ a cluster sample[9].

Intra-cluster correlation varies within a study and depends on the outcome under analysis[1,4,6]. The intra-cluster correlation of the same outcome may also vary across studies depending on the primary sampling unit, and whether outcomes are reported as prevalence rates or modeled in association with other variables[1,4]. Researchers need reliable estimates of the intra-cluster correlations, specific to the primary sampling unit and selected outcomes of interest when making sample size calculations. These estimates will assist in deciding the trade off between cluster number and cluster sub-sample size in a study design[10]. There is however, little published on the estimated intra-cluster correlation coefficients in the Australian context, especially for primary health surveys where the health practitioner is the primary sampling unit.

### Research questions

In one Australian study Carlin and Hocking[1] examined the intra-cluster correlation in two cross-sectional cluster surveys of school children that used the school as the primary sampling unit. The researchers observed that design effects for sociodemographic variables were larger than for morbidity related variables. Furthermore intra-cluster correlation was greater for descriptive outcomes such as prevalence estimates, means and proportions than for measures of association between variables such as regression coefficients and odds ratios. We wanted to examine whether these patterns could be generalised to other large cluster survey studies in the primary care setting.

This paper reports some of the intra-cluster correlations observed in the Bettering the Evaluation and Care of Health (BEACH) program, a large cross-sectional survey of general practice patient encounters in Australia, where a random sample of general practitioners was used as the primary sampling unit.

The BEACH study draws a new random sample of Australian general practitioners (GPs) each year, and this provided an opportunity to assess the stability of intra-cluster correlation coefficients across successive samples. If a population is re-sampled using the same cluster survey design, will the intra-cluster correlation coefficient for a particular outcome be the same across samples? This analysis takes an applied approach, examining the observed intra-cluster correlations for a range of demographic, morbidity and treatment outcomes.

## Methods

The BEACH program is a continuous study of general practice activity commenced in 1998. The BEACH method is described in detail elsewhere and a brief summary is reported below[11].

### Cluster sample design

A random sample of approximately 1,000 general practitioners (GPs) is drawn each year from the Health Insurance Commission's sampling frame of the population of GPs in Australia. The GP population is randomly ordered into a list and GPs are recruited sequentially from the list, with re-randomisation of the sampling frame every three years[11,12]. GPs are sampled without replacement and have one chance of selection over three years. Sampling is continuous across the year, with around 20 GPs participating in the study in any one week. Each GP completes details of 100 consecutive patient encounters. The GP is the primary sampling unit (PSU), while the primary unit of inference is the patient encounter.

### Data elements

A single page encounter form contains elements including:

• Patient age and sex.

• Whether English was the main language spoken at home.

• Whether the patient holds an Australian health care concession card.

• The problems managed by the GP at the encounter (up to four problems per encounter).

• Treatments received at the encounter, including medications, other procedures, referrals and orders for pathology and imaging tests.

Although sample weights are calculated each year for population estimates[13], the outcomes reported in this paper are unweighted to allow us to calculate estimates of the intra-cluster correlation based on the observed variance in the sample data.

### Descriptive outcomes

Descriptive outcomes were defined as rates, means and percentages of single variables, e.g. mean age, per cent of encounters with female patients, per cent of encounters where at least one respiratory problem was managed. BEACH samples the GP-patient encounter, not independent patients. If a patient returns to the GP in the sampling period then that patient contributes two (or more) encounters to the sample. Therefore BEACH estimates are not true "prevalence" rates because the denominator, the population of GP-patient encounters, is many times larger than the population of all general practice patients. To avoid misunderstanding in this paper we have used the term "descriptive" rather than "prevalence" to report single variable estimates and their accompanying intra-cluster correlation coefficients. Descriptive rates are interpreted for example as "Proportion of patients at encounter who are female".

#### Demographic variables

Demographic variables include patient sex, patient age, whether the patient held a health care concession card and whether the main language spoken at home was not English.

#### Morbidity variables

Problems were classified using the International Classification of Primary Care (ICPC-2)[14]. The upper level of ICPC-2 classifies problems according to the body system involved, for example skin problems, respiratory problems, cardiovascular problems and problems of the digestive system etc. There are an additional three chapters for psychological problems, social problems and problems of a general or unspecified nature. Morbidity estimates are expressed as the percent of patient encounters where at least one problem from the chapter was managed. The total number of problems managed by the GP at the encounter was also included as an outcome.

#### Treatment outcomes

Treatment outcomes included the proportion of encounters that resulted in at least one medication, the proportion that received at least one referral, the proportion that received at least one order for an imaging test and the proportion receiving at least one order for a pathology test.

### Association outcomes

Intra-cluster correlation coefficients were calculated for associations between variables using logistic regression e.g.: the effect of patient age (predictor) on the rate of cardiovascular problems (outcome).

### Design effect

Obtaining the sample size for cluster designs involves calculating the sample size under the assumption of simple random sampling and then inflating the number of observations to allow for the design effect of the cluster sample. The design effect (Deff) of an outcome has been defined as the ratio of the variance taking into account the cluster sample design and the variance of a simple random sample (srs) design with the same number of observations[1].

Deff = Variance_(clustersample)_/Variance_(srs)_

Intra-cluster correlations and their standard errors for the outcome variables were calculated using the method described by Carlin & Hocking[1]. Specifically STATA 7 was used to calculate the design effects using the "survey estimator" procedures, which were purposefully designed to analyse complex survey data. STATA 7 calculates the design effect directly from the ratio of the estimated variances[15].

The intra-cluster correlation coefficient (ICC) was then calculated from the design effect using the formula:

*ICC *= (*Deff *- 1)/(*k *- 1)

and the approximate standard error (SE) of the intra-cluster correlation was calculated using the formula[1,6]:



where *m *= number of clusters, *k *= mean number of observations per cluster.

The intra-cluster correlations and respective 95% confidence intervals for the second BEACH year sample from the period April 1999 to March 2000 were compared against those in the year 5 sample (April 2002 to March 2003) to assess whether the intra-cluster correlations were consistent across samples over time. All calculations specified the GP as the primary sampling unit.

## Results

From April 1999 to March 2000, 1,047 GPs were recruited, recording a sample of 104,700 patient encounters. From April 2002 to March 2003, 1,008 GPs were recruited and 100,800 encounters recorded. Table 1 shows the age and sex distribution of the two samples of GPs compared with the sampling frame of the population of Australian GPs in the year April 2002 to March 2003[13]. The two GP samples were comparable to the GP population in terms of distribution by age, sex and state.

**Table 1:** Comparison of GP participants and all active recognised Australian GPs.

	**BEACH April 1999–March 2000 % (95%CI) (N = 1,047)**	**BEACH April 02–March 03 % (95%CI (N = 1,008)**	**Australian GPs April 02 to March 03 % (N = 17,884)[13]**
**Males**	69.6 (66.8,72.4)	64.8 (61.8,67.7)	66.8
**Age group**			
<35	8.4 (6.7,10.1)	7.3 (5.7,9.0)	9.7
35–44	32.4 (29.6,35.3)	26.6 (23.9,29.3)	25.1
45–54	32.4 (29.6,35.3)	35.2 (32.3,38.2)	33.1
55+	26.7 (24.1,29.4)	30.9 (28.0,33.7)	32.0
**State**			
NSW	37.4 (34.5,40.4)	39.6 (36.7,42.7)	33.6
Victoria	20.1 (17.7,22.5)	18.8 (16.4,21.3)	24.5
Queensland	20.2 (17.8,22.6)	21.2 (18.7,23.8)	18.5
South Australia	9.1 (7.3,10.8)	6.2 (4.7,7.6)	8.7
Western Australia	8.8 (7.1,10.5)	8.9 (7.2,10.7)	9.5
Tasmania	2.4 (1.5,3.3)	2.8 (1.8,3.8)	2.9
ACT	1.1 (0.5,1.8)	1.4 (0.6,2.0)	1.5
NT	0.9 (0.3,1.4)	1.1(0.4,1.7)	0.8

The two samples of patient encounters were similar in terms of demographics (Table 2) In the year 1999–00, 59.0% of encounters were with female patients compared with 59.3% in 2002–03. The samples were comparable in terms of the mean age of patients, the proportion of health care card holders, and encounters with patients from a non-English speaking background.

**Table 2:** Descriptive parameters of demographic, morbidity and treatment variables with design effects (Deff), intra-cluster correlation coefficients (ICC) and standard errors of ICC (SE) for sample year April 1999 to March 2000 (N = 1,047 general practitioners): compared with ICC and SE for sample April 2002 to March 2003 (N = 1,008 GPs).

	**1999–2000 (N = 1,047 GPs)**			**2002–2003 (1,008 GPs)**	
	
**Parameter**	**Estimate(SE)**	**Deff ^(*a*)^**	**ICC(SE)**	**Estimate**	**ICC(SE)**
**Demographics**					
Sex (% female)	59.0 (.39)	6.4	.055 (.003)	59.3	.066 (.003)
Age (years) – mean	44.5 (.31)	16.6	.159 (.006)	45.4	.153 (.006)
Holds health care card (%)	40.1 (.70)	21.4	.206 (.007)	42.7	.209 (.008)
Patient language ^(*c*) ^(%)	7.0 (.53)	45.6	.451 (.011)	8.8	.423 (.011)
**Morbidity**					
Number of problems (per 100 encounters)	149.5 (.86)	13.6	.127 (.005)	148.7	.141 (.006)
*Problem by ICPC-2 chapter^(b)^*					
Cardiovascular (%)	15.2 (.29)	6.7	.057 (.003)	15.3	.056 (.003)
Respiratory (%)	21.0 (.26)	4.2	.032 (.002)	19.0	.040 (.002)
Psychological (%)	10.6 (.25)	6.8	.059 (.003)	10.6	.061 (.003)
Endocrine/Metabolic (%)	8.8 (.18)	4.2	.032 (.002)	10.1	.031 (.002)
Blood (%)	1.7 (.08)	3.7	.027 (.002)	1.4	.007 (.001)
Digestive (%)	9.6 (.12)	1.8	.008 (.001)	9.7	.010 (.001)
Eye (%)	2.8 (.06)	1.5	.005 (.001)	2.6	.003 (.001)
Musculoskeletal (%)	16.3 (.23)	4.1	.032 (.002)	16.5	.045 (.002)
Skin (%)	16.1 (.19)	2.7	.017 (.001)	15.9	.042 (.002)
General unspecified (%)	14.0 (.22)	4.3	.034 (.002)	15.8	.043 (.002)
**Treatment (% of encounters)**					
Any medications	67.0 (.37)	6.6	.056 (.003)	64.4	.068 (.003)
Any referrals	11.2 (.20)	4.1	.031 (.002)	12.0	.033 (.002)
Any pathology tests ordered	14.7 (.26)	5.8	.048 (.002)	16.0	.046 (.002)
Any imaging tests ordered	6.9 (.15)	3.8	.028 (.002)	7.8	.029 (.002)

^(*a*) ^Average number of observations per cluster *k *= 100, except age (*k *= 99.2) and sex (*k *= 98.8).

^(*b*) ^Per cent of encounters where at least one problem from the chapter was managed.

^(*c*)^Patient speaks a language other than English at home.

### Descriptive ICCs (March 1999–April 2000)

#### Demographics

For descriptive estimates of demographic variables the intra-cluster correlation ranged from 0.055 for sex of patient at encounter to 0.451 for language spoken by the patient at home. (Table 2). With a standard cluster size of 100 encounters this produced design effects ranging from 6.4 for patient sex to 45.6 for non-English speaking background.

#### Morbidity (ICPC body chapter)

For descriptive estimates of the management rates of morbidity problems, the intra-cluster correlations ranged from 0.005 for estimates of eye problems to 0.059 for estimates of psychological problems, with design effects of between 1.5 and 6.8 respectively.

#### Treatments

The intra-cluster correlation coefficients for treatments received ranged from 0.028 for any imaging tests ordered to 0.056 for any medications.

### Association ICCs

For bivariate relationships between an outcome and predictor, the association ICCs were considerably smaller than the descriptive ICCs (Table 3). This pattern was observed for both demographic and morbidity outcomes. When analysing the association between holding a health care card and other demographic variables, the ICCs ranged from 0.012 for patient sex to 0.128 for language background (Table 3), which were smaller than for the descriptive estimate of the percentage holding a health care card (Table 2).

**Table 3:** Associations between demographic and morbidity variables, measured as odds ratios, with design effect (Deff) and intra-cluster correlation coefficients (ICC) with standard errors (SE) for sample year April 1999 to March 2000 (N = 1,047 general practitioners): and ICC and SE for sample April 2002 to March 2003 (N = 1,008 GPs).

		**1999–2000 (N = 1,047 GPs)**			**2002–2003 (1,008 GPs)**
Outcome*	**Predictor**	**Odds Ratio**	**Deff ^(*a*)^**	**ICC (SE)**	**ICC (SE)**
**a) Demographic**					
Patient holds health care card	Female patient	1.07	2.2	.012 (.001)	.018 (.001)
	Age (years)	1.03	6.5	.056 (.003)	.073 (.003)
	Patient language^(*b*)^	1.26	13.7	.128 (.005)	.114 (.005)
Patient language^(*b*)^	Female patient	0.94	3.7	.028 (.002)	.024(.001)
	Age (years)	1.00	11.2	.104 (.004)	.098 (.004)
**b) Morbidity Chapters**					

Cardiovascular	Female patient	0.90	1.3	.003 (.001)	.004 (.001)
	Age (years)	1.05	2.1	.011 (.001)	.017 (.001)
	Holds health care card	2.55	2.4	.014 (.001)	.018 (.001)
	Patient language^(*b*)^	1.17	5.2	.042 (.002)	.034 (.002)

Respiratory	Female patient	.86	1.3	.003 (.001)	.003 (.001)
	Age (years)	.99	2.5	.015 (.001)	.022 (.001)
	Holds health care card	.89	2.1	.011 (.001)	.017 (.001)
	Patient language^(*b*)^	1.17	2.9	.020 (.001)	.025 (.002)

Psychological	Female patient	1.13	2.2	.013 (.001)	.008 (.001)
	Age	1.01	3.3	.024 (.001)	.022 (.001)
	Holds health care card	1.89	2.4	.014 (.001)	.020 (.001)
	Patient language^(*b*)^	.74	5.0	.040 (.002)	.026 (.002)

Endocrine/metabolic	Female patient	.95	1.6	.006 (.001)	.004 (.001)
	Age	1.03	2.1	.011 (.001)	.012 (.001)
	Holds health care card	1.63	2.4	.014 (.001)	.009 (.001)
	Patient language^(*b*)^	1.58	3.3	.023 (.001)	.019 (.001)

* Each predictor is fitted alone, each line represents a separate model.

^(*a*)^Average number of observations per cluster *k *= 100, except age (*k *= 99.2) and sex (*k *= 98.8).

^(*b*)^Patient speaks a language other than English at home.

When analysing the association between cardiovascular problems as the outcome and selected demographic variables, the ICCs ranged from 0.042 (patient language as the predictor) to 0.003 (patient sex as the predictor)(Table 3) compared with the larger ICC of 0.057 when describing the rate of cardiovascular problems (Table 2).

### Comparison of year 2 (April 1999 to March 2000) and year 5 (April 2000 to March 2003)

**Figure 1:**
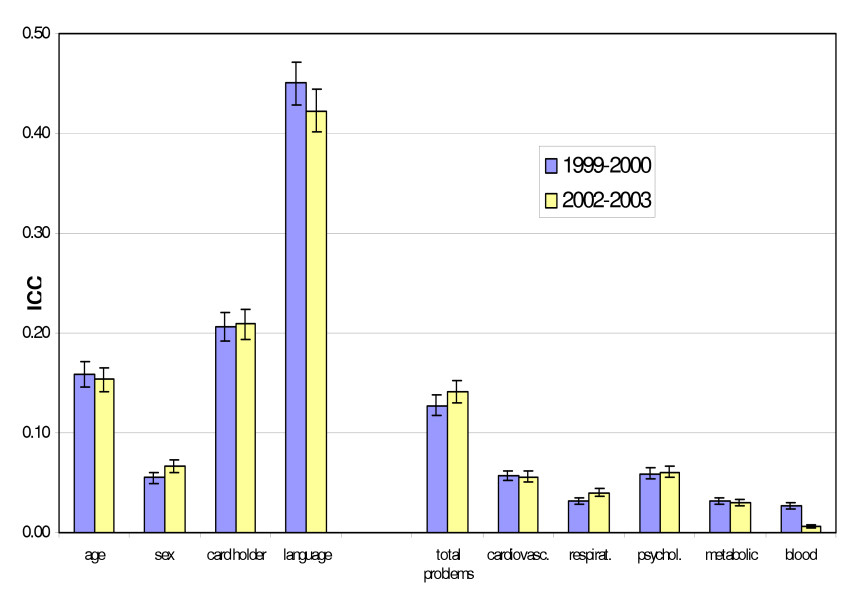
Intra-cluster correlation(ICC) and 95% confidence intervals for descriptive and morbidity outcomes in two BEACH samples, April 1999–March 2000 (N = 1047 GPs) and April 2002–March 2003(N = 1008 GPs) * Total problems = the number of problems managed at the current encounter.

For descriptive outcomes the intra-cluster correlations for year 2 and year 5 samples there was consistency in the patterns of ICCs across samples. (Table 2 and Figure 1). One exception was for the management of problems related to the blood system, where the descriptive ICC in 1999–00 was 0.027 (95% CI: 0.024–0.030), three times that observed in 2002–03 (0.007, 95% CI: 0.005–0.008). This was influenced by one GP in the 1999–00 sample who managed blood-related problems at more than 50% of encounters. When this GP was removed, the descriptive ICC for blood related problems in 1999–00 was 0.011 (95%CI: 0.009–0.013), much closer to the ICC observed in 2002–03.

**Figure 2:**
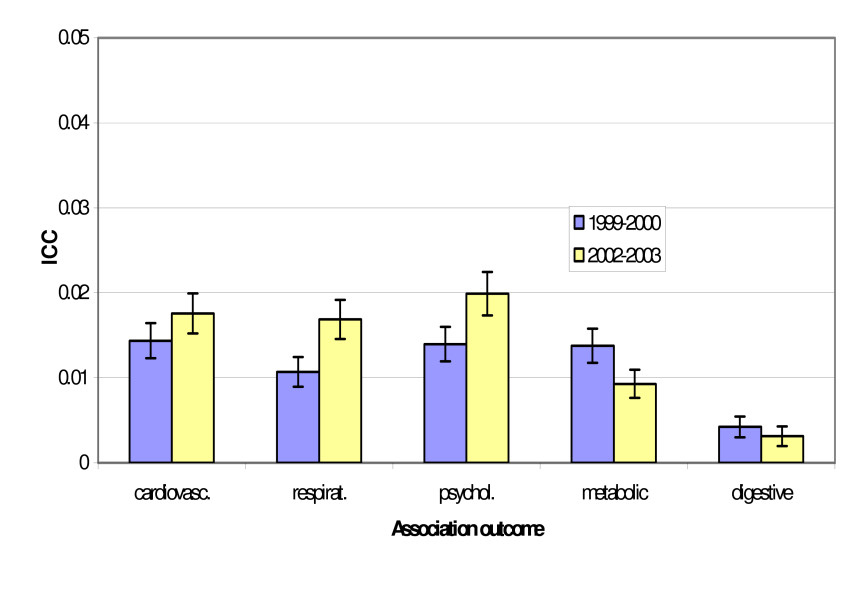
Intra-cluster correlation (ICC) and 95% confidence interval for association between morbidity outcomes with health care card status as predictor in two BEACH samples, April 1999–March 2000 (N = 1,047 GPs) and April 2002–March 2003 (N = 1,008 GPs)

The intra-cluster correlation for associations between morbidity outcomes and demographic predictors are shown in Table 3 and Figure 2. Although the intra-cluster correlations for associations between variables across each year were statistically significantly different for some outcomes, in these instances the ICCs were very small and the difference between samples was less than 0.01.

## Discussion

The pattern of intra-cluster correlation and design effects observed in the BEACH study agree with Carlin and Hocking's observations in other cluster sample surveys[1]. Generally we found that sociodemographic variables had larger intra-cluster correlation coefficients than morbidity or treatment variables and outcomes fitted with explanatory variables had smaller intra-cluster correlation coefficients than outcomes reported as descriptive rates. Therefore when designing cluster sample surveys, the effect of the intra-cluster correlation on power calculations, depends on whether the main outcomes of interest are demographic or morbidity variables, and whether the main aims of the study are descriptive or predictive[1].

We further demonstrated that for a large range of variables the size and patterns of intra-cluster correlation coefficients for particular outcomes were mostly consistent over different sample periods. This indicates that intra-cluster correlation is quite stable when re-sampling a population using the same primary sampling unit, where the number of clusters is sufficiently large. This repeatability demonstrates the validity of using published intra-cluster correlation coefficients to predict intra-cluster correlation in future studies of similar design.

Precision can be an issue for estimating intra-cluster correlation, especially for studies with a small number of clusters[10]. The large number of clusters in this study gave good precision in the estimated intra-cluster correlation coefficients[10]. There are no other published studies in general practice in Australia with such a large sample of clusters and a large balanced sample of observations per cluster, thus estimating intra-cluster correlation with a high degree of precision.

The BEACH study also has the advantage of being a nationwide survey of general practice where the generalisability to Australian general practice has been well-described[11]. Most research in primary care in Australia is done through general practice, so estimating the intra-cluster correlation for a range of outcomes is important for future researchers who intend to use the GP as the primary sampling unit. The good representation of general practice in the BEACH study, the large sample of clusters and the large cluster size, allow the intra-cluster correlation coefficients reported here to be generalisable to other general practice surveys. These reported intra-cluster correlation coefficients are also likely to be useful for intervention studies that use the GP as the unit of randomisation[1].

Treatments received at the encounter are outcomes that arise as a result of the GP-patient interaction. Treatments are directly related to GPs' behaviour and so might be expected to be highly correlated within clusters. However we found that the intra-cluster correlation coefficients for medications, referrals, imaging and pathology orders were of a similar order to those for health problems managed.

The difference across samples in the intra-cluster correlation coefficients for the management of blood system problems indicates that, even in large samples, intra-cluster correlation may be influenced by GPs in the sample who specialise in particular areas of health.

Demographic variables are collected in the BEACH study for the purpose of understanding health status and health service use and these variables are likely to be correlated to a patient's choice of GP. Furthermore a patient can be sampled more than once if they return to the GP during the survey period. Therefore the intra-cluster correlation estimated for demographic variables may be larger than those that have been reported in community based surveys[1,8].

## Conclusions

As with cluster randomised trials, researchers in primary health care need access to a range of estimates of intra-cluster correlation for the successful planning of cluster survey study designs. We have reported relatively stable intra-cluster correlation coefficients for a range of outcomes across two independent random samples in a large-scale representative survey of general practice in Australia. The demonstrated precision and reliability of the estimated intra-cluster correlations indicate that these coefficients will be useful for calculating sample sizes in future general practice surveys that use the GP as the primary sampling unit.

## Abbreviations

GP: General Practitioner

ICC: Intra-cluster correlation coefficient

## Competing interests

The author(s) declare that they have no competing interests.

## Authors contributions

SK conceived the research questions, undertook the analysis and wrote the main draft of the manuscript. PC participated in formulating the research questions and the design of the analysis, undertook a literature search and assisted in the writing of the main draft and subsequent revisions of the manuscript.

## Pre-publication history

The pre-publication history for this paper can be accessed here:


